# Diversion Detection in Small-Diameter HDPE Pipes Using Guided Waves and Deep Learning

**DOI:** 10.3390/s22249586

**Published:** 2022-12-07

**Authors:** Abdullah Zayat, Mohanad Obeed, Anas Chaaban

**Affiliations:** School of Engineering, University of British Columbia, 1137 Alumni Ave, Kelowna, BC V1V 1V7, Canada

**Keywords:** high-density polyethylene (HDPE), ultrasonic-guided waves (UGWs), Zadoff–Chu sequence, deep neural network (DNN), convolutional neural network (CNN), recurrent neural network (RNN), long-short term memory (LSTM), piezoelectric, structural health monitoring (SHM)

## Abstract

In this paper, we propose a novel technique for the inspection of high-density polyethylene (HDPE) pipes using ultrasonic sensors, signal processing, and deep neural networks (DNNs). Specifically, we propose a technique that detects whether there is a diversion on a pipe or not. The proposed model transmits ultrasound signals through a pipe using a custom-designed array of piezoelectric transmitters and receivers. We propose to use the Zadoff–Chu sequence to modulate the input signals, then utilize its correlation properties to estimate the pipe channel response. The processed signal is then fed to a DNN that extracts the features and decides whether there is a diversion or not. The proposed technique demonstrates an average classification accuracy of 90.3% (when one sensor is used) and 99.6% (when two sensors are used) on $\frac{3}{4}$ inch pipes. The technique can be readily generalized for pipes of different diameters and materials.

## 1. Introduction

Pipelines are extensively utilized for oil and natural gas transfer. However, one of the main concerns with pipelines is leakage, with incidents occurring on a regular basis [1]. In addition to human errors, many other factors contribute to pipeline leakage, including external impacts, material faults during manufacturing time, environmental corrosion, internal erosion, ground surface movements, and inappropriate maintenance [2]. Early detection of structural degradation due to such factors is required to maintain structural safety and integrity to lower the likelihood of a catastrophic failure. Thus, the development of robust and cost-effective diagnosis techniques to ensure the structural safety of pipes has long been of great importance [1].

Many works in the literature have studied diagnosis methods (nondestructive testing) for steel pipes, such as radiography, conventional ultrasonic testing, acoustic emission, visual inspection (including thermal imaging and laser scanning)and ground penetrating radar [2,3,4,5,6,7]. However, less work has been conducted on the diagnosis of high-density polyethylene (HDPE), which is commonly used in residential natural gas service lines. Nonetheless, the diagnosis of HDPE pipes is important for several reasons including leak detection and pipe integrity monitoring [8].

Gas diversions are carried out illegally by using methods to obstruct the flow of natural gas through the pipe and installing a T-fitting that redirects natural gas to an unmetered location for unmeasured consumption. This tampering with the pipe poses many risks since it is unrecorded, violates pipeline quality standards, and can lead to potential leaks and possibly explosions, consequently posing significant risk to public safety, property, and the environment in the vicinity of altered gas lines. Such diversions have been discovered in the past through word of mouth, leaks, or unexpected encounters with an unrecorded natural gas pipe in a construction site [9,10,11,12,13].

Therefore, effective and timely techniques for diversion detection are required to avoid such risks. Given the concealed nature of below-grade pipes, it is very challenging to inspect them. Existing solutions include ground penetrating radar and endoscope cameras [14,15], which are both invasive and expose inspectors to potential risk from the suspects. As a result, it is better to use noninvasive methods to inspect pipes.

In this paper, we investigate the possibility of using ultrasonic-guided waves (UGW) to inspect HDPE pipes. UGW sensing offers fast screening, which enables the inspection of buried, insulated, and underwater pipelines using ultrasonic sensors, while providing a larger range of inspection than traditional ultrasonic testing because it uses the structure of the pipe itself as a waveguide [16]. In UGW, signals are generated using ultrasonic transducers that are coupled to the surface of the pipe so that waves propagate through the pipe. UGW sensing has gotten a lot of attention from the industry because of its long-range inspection capabilities from a single test location e.g., they can inspect more than 100 m of pipelines from a single test location [5]. Although many works have studied the inspection of metallic structures using UGWs techniques [6,7,17], the use of UGW sensing to inspect nonmetallic structures such as HDPE pipelines is still in its early stages [18].

The transmission and reception of ultrasonic guided-waves is commonly realized using piezoelectric or magnetoresistive sensors. Due to the low cost of piezoelectric transducers and their wide availability, they have attracted most attention [19]. Such sensors are commonly arranged in an array which generate desired wave modes for sensing applications [6]. The reflected signals are then received and processed to reveal potential structural defects [6,7]. In many cases, such defects are very difficult to detect using classical techniques and/or visual inspection of the received signal [20], which is especially the case with bent pipes and when using simple transducers (both of which are relevant when inspecting residential HDPE pipes). To tackle this issue, machine learning (ML) and deep neural networks (DNN) have been proposed and utilized to extract hidden useful information from the received signals, leading to improved detection accuracy with low computational complexity [21,22].

A recent review article [17] addressed the use of UGW and ML for defect detection. Some examples include structural health monitoring using piezoelectric sensors and ensemble learning by consensus using support vector machines [23] and using DNNs [24], defect sizing in pipes using ML algorithms and simulated and experimental data [25], estimating transducer-to-defect distance using the convolutional neural network (CNN) [26], assessing the degree of damage to a thin metal plate using wave-field measurements and DNN [27], and estimating the impact site of a steel ball on an aluminum plate using ML [28], among others. These works showed that high accuracy sensing is possible using their proposed methods. Recently, Sun et al. [29] studied a technique to evaluate plate damage using UGW and DL methods, including CNN, recurrent neural networks (RNN), and residual neural network called GFresNet, showing up to 95% accuracy using GFresNet.

The objectives of this paper are as follows:

- To propose, design, and test piezoelectric transducer arrays, electronic circuits, signal designing and processing, and DNN models required to develop a DNN-based UGW detection scheme.
- To implement and evaluate a DNN-based UGW detection scheme, for detecting diversions in HDPE pipes.

To this end, we design two clamps containing an array of piezoelectric transmitters and receivers to enable transmitting waves through the HDPE pipes. To develop the transmitter and receiver signal processing, we employ Zadoff–Chu sequences (due to their desired correlation properties [30]) at the transmitter side and correlation methods at the receiver side to estimate the pipe’s impulse response. These signals are designed in a computer, which is interfaced with the transmitters and receivers through a waveform generator, an oscilloscope, and the necessary driving circuits. To classify the received correlation signals, we develop a DNN architecture that extracts features from the correlation signal and use these features to detect a diversion. The DNN consists of a CNN that extracts features from segments of the correlation signal, followed by a long short-term memory (LSTM) network that extract features from temporal correlations between segments, followed by a multi-layer perceptron that makes a decision. We train and test the DNN using signals from experiments on control pipes and pipes with diversions, and show that the developed method is able to classify control and diversion samples with an accuracy of 90.3% when one receiving sensor is used, and 99.6% when two sensors are used.

The details of the proposed method are described in the rest of the paper. Section 2 presents the system specifications and problem statement. The signal design and processing techniques are presented in Section 3. Empirical data collection is described in Section 4, and the proposed DNN architecture is illustrated in Section 5. The results are presented in Section 6, and the paper is concluded in Section 7.

## 2. System Specifications

**Figure 1:**
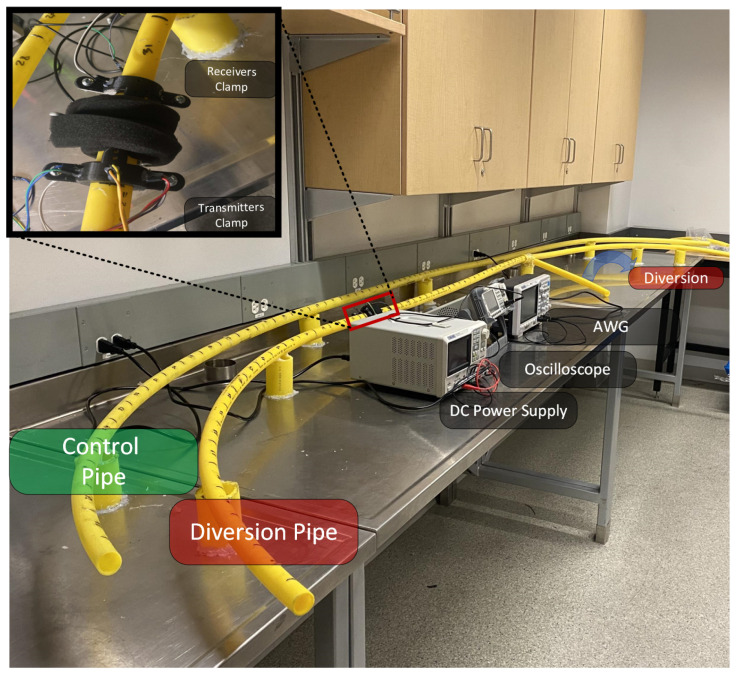
Pipe samples: the pipe on the right has a diversion (tee-fitting), whereas the one on the left does not (control pipe).

We consider HDPE pipes with an outer circumference of 26.7 mm and a wall thickness of 2 mm ($\frac{3}{4}$ inch pipe), which are commonly used for transporting and distributing natural gas to households. For the purpose of diversion detection, we consider two types of pipes as shown in Figure 1: a control pipe without a diversion, and a pipe with a diversion, i.e., a tee-fitting installed along the pipe. The lengths of the considered pipes are 1, 2, 3, and 5 m, with a diversion installed at 0.6, 1.2, 1.5, and 3 m from one end of the pipe for diversion samples, and with nothing installed on the control samples. The length of the pipe extending from the tee-fitting is 30 cm.

The goal is to detect the diversion using UGW and machine learning. To this end, piezoelectric transducers are used to transmit/receive ultrasonic waves. For UGW testing, a range of the operation frequency between 20 kHz and 100 kHz is widely used [31], which was considered when selecting the transducers. We opted for transducers with 40 KHz frequency due to its availability in the market with proper dimensions. Moreover, utilizing 40 KHz can help in mitigating the attenuation effect, which increases the detection range in return [32]. These transducers generate an electrical signal in response to a sensed mechanical signal (propagating through the pipe), which is known as the piezoelectric effect, or generate a mechanical signal (to be sent through the pipe) in response to a driving electrical signal, which is known as the reverse piezoelectric effect.

**Figure 2:**
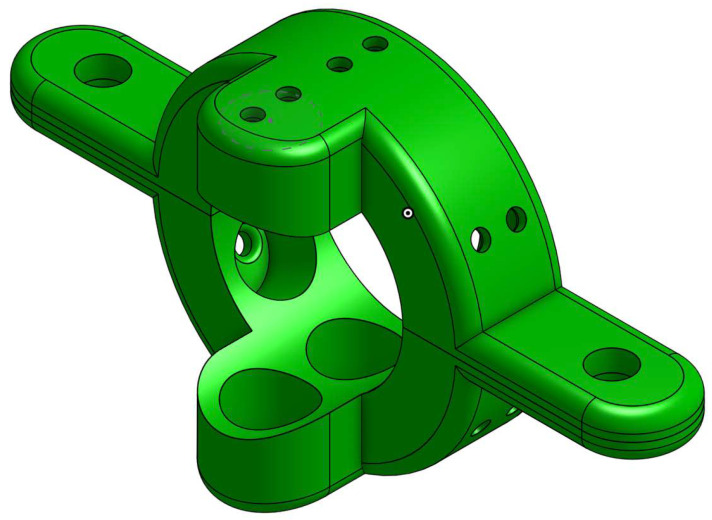
Clamp design: The clamp has a total of eight slots, six of which form a ring where transducers can be placed 60 degrees apart, and two additional slots which are 180 degrees apart for deploying additional transducers. This clamp was modified from the original design in [33].

The transmitters and receivers are placed in two clamps (Figure 2) which are designed to fit the pipe and to hold the transducers. Placing multiple transducers in each clamp can aid in improving signal generation (transmitted power and modes) as well as signal detection. For the purpose of this paper, the transmitter clamp was equipped with six transmitters, while the receiver clamp was equipped with two receivers, distributed evenly around the circumference of the pipe.

**Figure 3:**
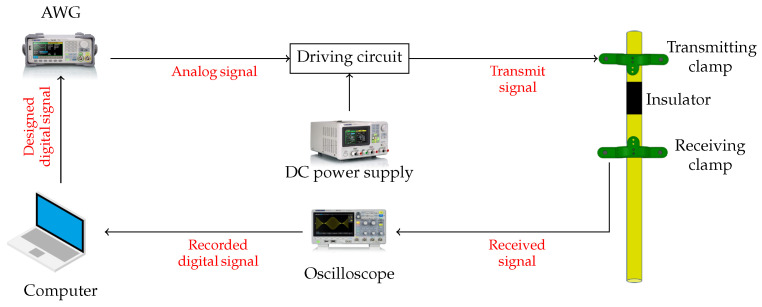
Experimental setup: The designed signal is transmitted via ultrasonic transmitters with the aid of an AWG (arbitrary waveform generator), a DC power supply, and a driving circuit, and received using ultrasonic receivers with the aid of an oscilloscope. Signal processing takes place in a computer.

To sense the pipe for a diversion, we use the system shown in Figure 3. First, the signal to be transmitted is designed in Matlab. Then it is sent to an arbitrary waveform generator (AWG, Siglent Technologies SDG2042X), which is interfaced with an Op-Amp driving circuit (2 KΩ and 10 KΩ resistors and a 741 Op-Amp), powered by a DC-power supply (Siglent Technologies SPD3303X-E), which in turn drives the ultrasonic transmitters (Murata Electronics MA40S4S). The transmitters generate a wave that propagates along the pipe’s circumference. The reflected signal is captured using the receivers (Murata Electronics MA40S4R) which are interfaced with an oscilloscope (Siglent Technologies SDS1104X-E) that records the signal. Finally, the recorded signal is sent to a PC for signal processing and analysis.

Due to the dispersion effect that takes place whenever an ultrasound signal travels through a solid object (in this case, the pipe), the wave will be decomposed into three distinct modes: flexural, torsional, and longitudinal modes [17]. These modes travel through the pipe at varying speeds depending on the mode. As a result, analyzing the received signal can be challenging, as the signal is likely to be a mix of these modes. Mode filtering/isolation is often used to improve the received signal quality [34]. However, to keep the process simple and to use all modes combined for diversion detection, we replace the classical signal processing techniques with a machine learning technique applied after correlating the received signal with the Zadoff–Chu sequence as explained next.

## 3. Signal Design and Processing

This section discusses the design of the transmitted signal and its benefits, in addition to the signal processing of the received signal, which is applied before feeding the signal into the DNN for classification. We provide a generic description of the scheme first, and then we specify the utilized parameters afterwards.

### 3.1. Transmitted Signal Design

To sense the pipe, we interpret the pipe as a channel whose impulse response can be used as a signature to determine the presence of a diversion or the absence thereof. Following this interpretation, we propose to transmit a training signal with good correlation properties in line with signals used for channel estimation, rather than a pulse as in [18,19,35]. There are several signals that can be used for this purpose, including maximum-length pseudo-noise sequences [36] and Zadoff–Chu sequences [30], which are known to aid in channel estimation as well as providing immunity against disturbances, such as noise and interference. These sequences have good auto-correlation properties, namely, their autocorrelation resembles a Dirac impulse. As such, when such a sequence is transmitted, correlating the received signal with the sequence itself yields the channel impulse response (within some distortion due to noise).

We opt for using a Zadoff–Chu sequence due to its desirable auto-correlation properties. A Zadoff–Chu sequence (named after Solomon A. Zadoff and D.C. Chu [30,37]) is a complex valued poly-phase sequence with constant amplitude and zero auto-correlation (CAZAC). A length *N* Zadoff–Chu sequence s[k], k=0,1,…,N−1, can be expressed as

(1) $$s\left[k\right]=e^{i\alpha_{k}},k=0,1,\ldots ,N-1,$$

where $\alpha_{k}$ is given by

(2) $$\alpha_{k}=\left\{\begin{array}{cc} \frac{M\pi k^{2}}{N} & \mathrm{if}\;N\;\mathrm{is}\;\mathrm{even},\\ \frac{M\pi k(k+1)}{N} & \mathrm{if}\;N\;\mathrm{is}\;\mathrm{odd}, \end{array}\right.$$

and *M* is coprime with *N*. To convert this to a time-domain signal that can be transmitted using the transducers, we use pulse shaping using a sinc function $\frac{\sin(\pi t/T_{c})}{\pi t/T_{c}}$, where $T_{c}$ is the pulse duration, sampled at a sampling rate of $f_{s}$ to obtain the signal

(3) $$\begin{array}{c} \tilde{s}\left[n\right]=\sum_{k=0}^{N-1}s\left[k\right]\frac{\sin\left(\pi \left(\frac{n}{f_{s}T_{c}}-k\right)\right)}{\pi \left(\frac{n}{f_{s}T_{c}}-k\right)}, \end{array}$$

where $n=0,1,\ldots ,NT_{c}f_{s}-1$.

The signal $\tilde{s}\left[n\right]$ is then modulated using a $f_{c}=40$ KHz carrier (the frequency of the transducer) to obtain the transmitted signal

$$x\left[n\right]=\tilde{s}\left[n\right]e^{j2\pi f_{c}n/f_{s}},$$

$n=0,1,\ldots ,NT_{c}f_{s}-1$ (note that we require $f_{s}>2f_{c}$ to avoid aliasing).

### 3.2. Received Signal Processing

The received signal of each receiver is first sampled at a sampling rate of $f_{s}$, and bandpass filtered using an infinite-impulse-response (IIR) bandpass filter centered at 40 KHz with a 3 dB bandwidth of 10 KHz to eliminate out-of-band noise and interference. The resulting signal can be modeled as

(4) y[n]=h[n]⊛x[n]+w[n],

where *n* is the time index, h[n] is the impulse response of the pipe, ⊛ is the convolution operator, and w[n] is noise. A general *K*-tap impulse response can generally be represented as $h\left[n\right]=\sum_{k=0}^{K-1}\beta_{k}\delta \left[n-d_{k}\right]$ for some *K*, where δ[n] is the Dirac delta pulse which equals 1 when n=0 and 0 otherwise, and $\beta_{k}\in R$ and $d_{k}\in N$ are the attenuation coefficient and the time delay of the *k*-th tap.

To estimate the channel response, we cross-correlate the received signal y[n] with the reference signal x[n] to obtain the following signal

(5) $$\begin{array}{cc} r[n] & =y\left[n\right]⊛x^{*}\left[-n\right] \end{array}$$

(6) $$\begin{array}{cc} \; & \;\;\;\;\;\;\;=h\left[n\right]⊛x\left[n\right]⊛x^{*}\left[-n\right]+\tilde{w}\left[n\right], \end{array}$$

where $\tilde{w}\left[n\right]=w\left[n\right]⊛x^{*}\left[-n\right]$ is the filtered noise. Due to the properties of the Zadoff–Chu sequence, where $s\left[k\right]⊛s^{*}\left[-k\right]\approx N\delta \left[k\right]$ [37], we have the following (note that while the circular correlation of the Zadoff–Chu sequence coincides with a Dirac pulse, the linear correlation is very close (but not equal) to a Dirac pulse):

$$x\left[n\right]⊛x^{*}\left[-n\right]=N\frac{\sin\left(\pi \left(\frac{n}{f_{s}T_{c}}\right)\right)}{\pi \left(\frac{n}{f_{s}T_{c}}\right)}e^{j2\pi f_{c}n/f_{s}}$$

where we replace the approximation by an equality for simplicity. This yields

(7) $$\begin{array}{cc} \;\;\;r[n] & \approx \sum_{k=0}^{K-1}\beta_{k}\delta \left[n-d_{k}\right]⊛N\frac{\sin\left(\pi \left(\frac{n}{f_{s}T_{c}}\right)\right)}{\pi \left(\frac{n}{f_{s}T_{c}}\right)}e^{j2\pi f_{c}n/f_{s}}+\tilde{w}\left[n\right] \end{array}$$

(8) $$\begin{array}{cc} \; & =\sum_{k=0}^{K-1}\beta_{k}N\frac{\sin\left(\pi \left(\frac{n-d_{k}}{f_{s}T_{c}}\right)\right)}{\pi \left(\frac{n-d_{k}}{f_{s}T_{c}}\right)}e^{j2\pi f_{c}\left(n-d_{k}\right)/f_{s}}+\tilde{w}\left[n\right] \end{array}$$

To eliminate the carrier, we then apply a Hilbert transformer and compute its magnitude to obtain the envelope of r[n] [38]. The resulting signal will have prominent peaks at time instants $d_{k}$ (as can be observed in (8)), which can be interpreted as a ’signature’ of the channel h[n]. It is expected that the signal transmitted through a control pipe will produce a signature that is different from the one produced from a diversion sample. This can be used to classify the pipes accordingly.

**Figure 4:**
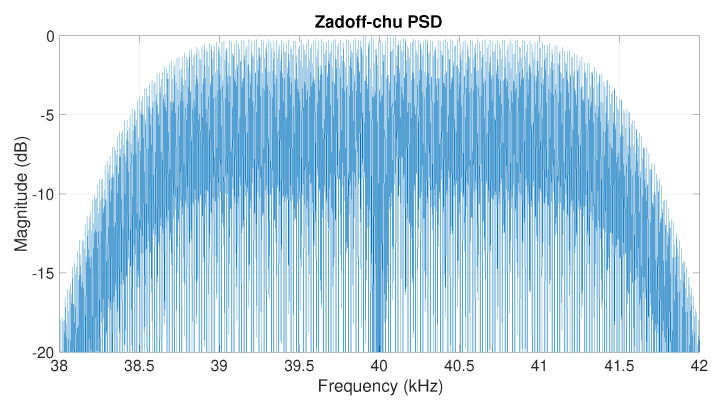
Power spectral density of transmitted Zadoff–Chu sequence (x[n]).

**Figure 5:**
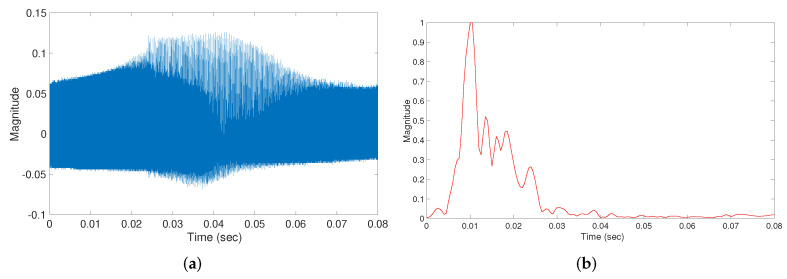
Received signal before and after processing. (**a**) Raw received signal y[n]. (**b**) Received signal before and after processing.

In this work, we use N=512 and M=1 to generate a Zadoff–Chu sequence, and we use $T_{c}=300$ μs and $f_{s}=1$ MHz. As seen in Figure 4, the resulting signal has a central frequency of 40 KHz and a 3-dB bandwidth of 3 KHz which can be transmitted using the selected transducers that have a bandwidth of 3.3 KHz. Figure 5 depicts the received signal y[n] in (4) and the envelope of the correlation signal in (8). Due to the low-frequency characteristics of this envelope, we down-sample it to 10 KHz. The resulting signal envelope, which has a total of 320 time samples, is used as an input for the proposed DNN.

Note that a similar process is applied to the signal received from each of the two sensors. Next, we describe our data collection procedure, which uses the signal processing explained above.

## 4. Data Collection

To train and assess a deep learning algorithm for detecting diversions, we collect a dataset of correlation envelope signals (similar to Figure 5b) using the experimental setup described in Section 2 and the signal processing in Section 3. We consider two types of pipes, namely control and diversion pipes. To diversify the dataset, we use pipes with lengths of 1, 2, 3, and 5 m, with diversions (in the diversion samples) at distances of 0.6, 1.2, 1.5, and 3 m from one end of the pipe, respectively.

For each pipe, we attach the clamps so that the distance between the transmitting and receiving clamps is 11 cm. Additionally, to guarantee consistent readings of the received signals, we make sure the transmitting and receiving clamps are oriented similarly. To do this, we mark the upper side of each clamp (see Figure 2), and we ensure that a line passing through the two marks is coplanar with the axis of the pipe.

Initially, the clamps are installed so that the transmitting (respectively receiving) clamp is 20 cm (respectively 31 cm) away from the beginning of the pipe. To collect a diverse set of measurements from this clamp position, we rotate the clamps with increments of 120 degrees while maintaining the alignment of the transmitting and receiving clamps. After collecting data from this position, the clamps are advanced by 4 cm, and the same process is repeated to collect more data. This process is repeated until the clamps are 20 cm away from the diversion on the diversion pipes, and at a similar position on the control pipes. For each position and rotation, 2 to 4 samples are collected from each of the two sensors.

**Figure 6:**
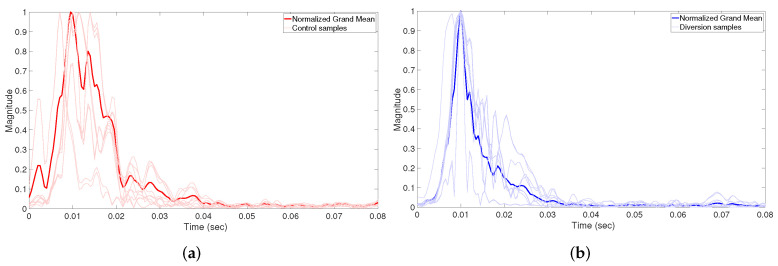
Correlation envelopes of diversion and control samples. Each sub-figure shows a plot of 40 samples selected randomly from the datasets, in addition to the mean of all samples in the datasets. (**a**) Diversion samples. (**b**) Control samples.

We collected a dataset of 2292 samples, 1006 of which were from control pipes and 1286 from diversion pipes. Figure 6 shows the correlation envelope of a number of samples from each class (diversion and control). The figure shows the presence of differences between the two classes, the most evident of which is the delay spread which is larger in diversion samples than in control samples. However, the figure also shows that it is not always reliable to use this difference to classify the pipes. Fortunately, a deep neural network (DNN) can be used to learn subtle differences between the samples to produce a more reliable classification. Thus, we train a DNN to classify the pipes based on the correlation envelope signals, which is detailed in the next section.

## 5. DNN Architectures

The traditional techniques that are usually employed in the area of non-destructive testing (NDT) to categorize flaws are prone to subjective influences, such as a person’s opinion [39]. In addition, since the received signals are one-dimensional, it is difficult to use them to precisely detect diversions by visual inspection. This challenge can be addressed by using an automated detection system based on a DNN to provide high-accuracy detection and avoid the manual detection drawbacks.

The first step in the proposed DNN is to learn features from the data. Various methods have been used in the literature to extract or learn features using time and frequency domain features [23,25], using wavelets and a CNN [26,40], or using pre-established standard time-series classification networks, such as long short-term memory recurrent networks (LSTMs) [41]. In this paper, we use CNN and LSTM layers to learn features from the correlation envelope signals. We investigate three types of DNN models, namely CNN-based models, LSTM-based models and CNN-LSTM-based models, all implemented in TensorFlow. These models were chosen based on their ability to extract shape features (CNN-based models), analyzing time-series signals (LSTM-based models) or combining both (CNN-LSTM-based models). In the following, we explain these models in details.

### 5.1. CNN Based Model

It can be seen from Figure 6 that the correlation envelope signals of the control sample have different shapes than those of the diversion samples. Therefore, we can use the signal shape to classify the pipe. Since our data consist of correlation envelop signals, and since a CNN is known for its accuracy when identifying shapes, it can also be used to extract shape features from a 1D signal as in the correlation envelope signal.

**Figure 7:**
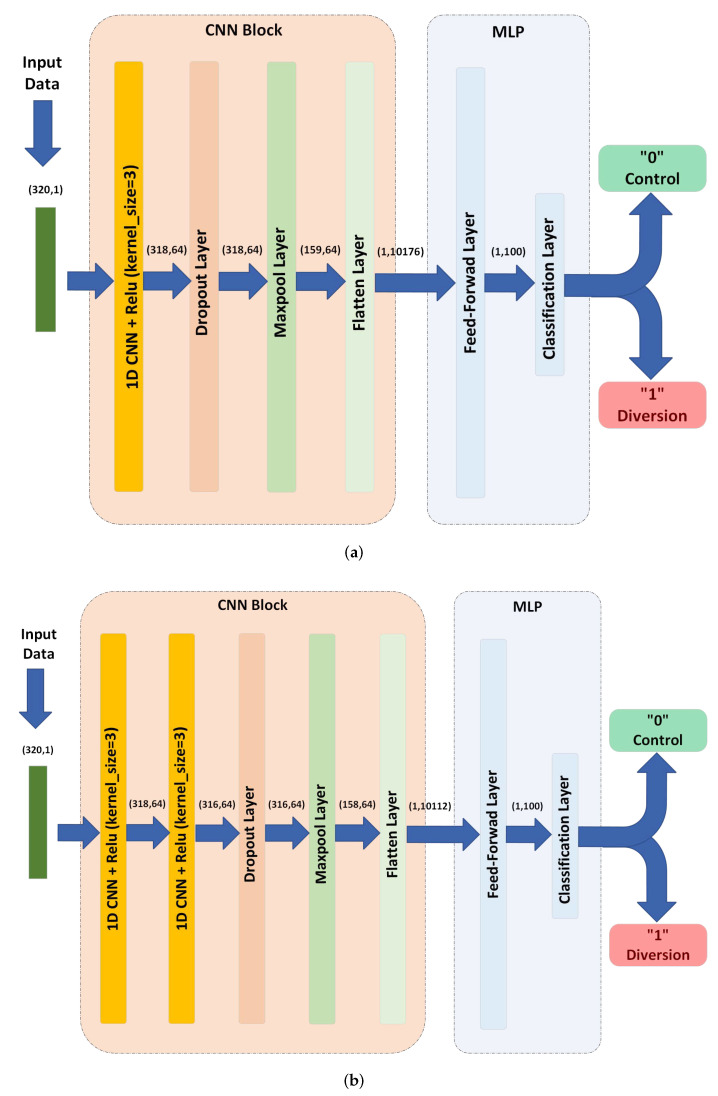
CNN-based models’ architectures. (**a**) CNN Model. (**b**) 2 Stacked CNN model.

A CNN-based model can contain one or multiple stacked CNN layers. In this paper, we consider one and two CNN layer models (CNN model and two stacked CNN model) with 64 filters in each layer as shown in Figure 7 (since it was noticed that increasing the number of layers/filters further does not improve accuracy). We describe the 2-layer architecture, noting that the 1-layer architecture can be described in an analogous way.

We use the correlation envelope signals, represented as vectors with dimension 320 × 1, as inputs to the first CNN layer which uses 64 filters (kernels) each with size 3×1. Each filter learns to extract only one feature from the input sequence. By applying this filter to the whole input (which is 320×1), this results in an output of size 318×1 for each filter. Since 64 filters are used, this process yields a 318×64 array of features as an output. This output is then fed to a rectified linear unit (ReLU) activation layer, which is commonly used in many neural networks, and it is known for its fast training and superior results.

The output of the first CNN layer is further processed by feeding it to another CNN layer. The propose of using a second layer is to extract composite features (features of features). As a result, the two layers combined extract features with complex patterns from the input signal. The second CNN layer operates similar to the first one, where 64 distinct filters of size 3×64 and a ReLU activation layer are used to obtain a 316×64 output array.

A dropout layer is used at the output of the second CNN as a regularization technique to prevent over-fitting. It randomly ’drops’ some neurons from the CNN (by setting their output to zero), which prevents the model from relying on specific features or neurons. In the training stage, the dropout layer randomly chooses a number of neurons to drop in each training iteration. The number of neurons is calculated based on a parameter called the drop out ratio, which varies from 0 (no dropout) to 1 (full dropout). We choose a ratio of 0.5 which means that 50 percent of the neurons are dropped at random (chosen since it led to the highest accuracy among a set of examined ratios). Note that, as this layer is a regularization layer, it is deactivated after the model is trained. This layer does not change the size of its input and thus its output is a 316×64 array.

A max pooling layer is often employed after a CNN to reduce the complexity through downsampling and to avoid data over-fitting. This is implemented by pooling a number of outputs into one output through choosing their maximum value. We used a pooling size of 2 and a stride of 2, which means that the output is the maximum value in non-overlapping windows of size 2 for each filter and segment. As a result, the output becomes a 158×64 array.

After the features are extracted using the previous steps, we need to convert these features into a one feature vector to prepare it for classification. This is achieved using a flatten layer that reshapes features of shape 158×64 into a vector of shape 1× 10,112. These features are then fed to the multilayer-perceptron layer, which will be explained in Section 5.4.

### 5.2. LSTM Based Models

**Figure 8:**
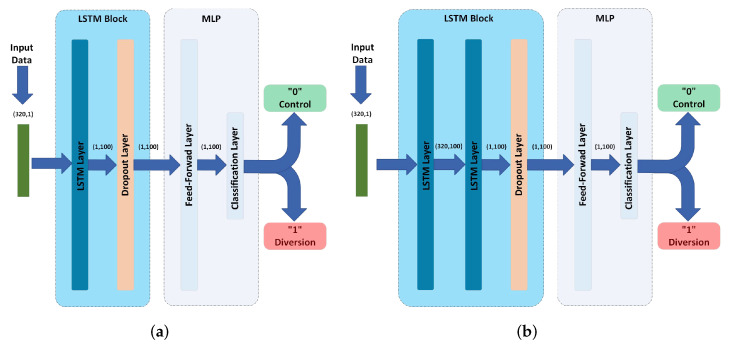
LSTM-based models’ architectures. (**a**) LSTM model. (**b**) 2 Stacked LSTM model.

An LSTM is a type of RNN which is used to extract features from sequential data. Unlike feed-forward neural networks, LSTMs include feedback connections to enable them to learn dependencies between time-correlated data points. In this paper, we examine two LSTM-based models, i.e., the LSTM model and 2 stacked LSTM model, as shown in Figure 8.

**Figure 9:**
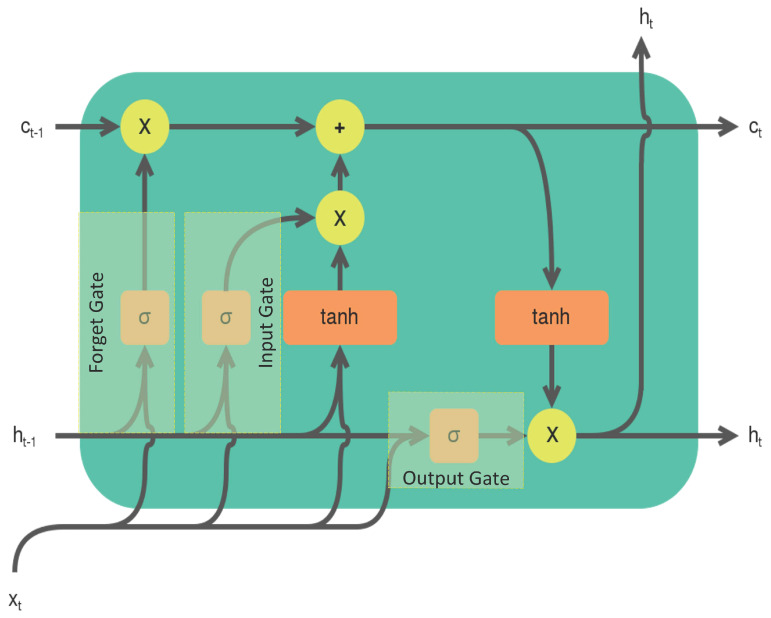
LSTM cell.

As shown in Figure 9, a typical LSTM cell consists of three gates: a forget gate, an input gate, and an output gate. It has an input vector $x_{t}$, a cell state $c_{t}$, and a hidden state $h_{t}$. The hidden state is computed from $c_{t}$, which is used as feedback. The output of the LSTM cell is the hidden state obtained after processing the whole sequence ${\left\{x_{t}\right\}}_{t=0}^{319}$.

The gates in the LSTM cell regulate the flow of information from one time step to the other. The forget gate uses the current input $x_{t}$ and previous hidden state $h_{t-1}$ to gate the previous cell state $c_{t-1}$. The input gate uses $x_{t}$ and $h_{t-1}$ to determine their contribution to the current cell state $c_{t}$. The output gate uses $x_{t}$ and $h_{t-1}$ to determine if the current cell state $c_{t}$ contributes to the current hidden state $h_{t}$ which will be fed back to the LSTM cell in the next time step. This mechanism enables the LSTM cell to extract one feature from the time series ${\left\{x_{t}\right\}}_{t=0}^{319}$ while considering the temporal dependencies between the time steps.

We utilize an LSTM layer that consists of 100 LSTM cells to extract 100 features from the input time series ${\left\{x_{t}\right\}}_{t=0}^{319}$ of dimension 320×1. This leads to an output of dimension 1×100.

To regularize the LSTM layers, we use a dropout layer with a dropout ratio of 0.5. This is only used while training and removed afterwards. It remains to use this information to produce a classification outcome, 0 for control and 1 for diversion, which is done using a multi-layer perceptron.

### 5.3. CNN-LSTM Based Models

**Figure 10:**
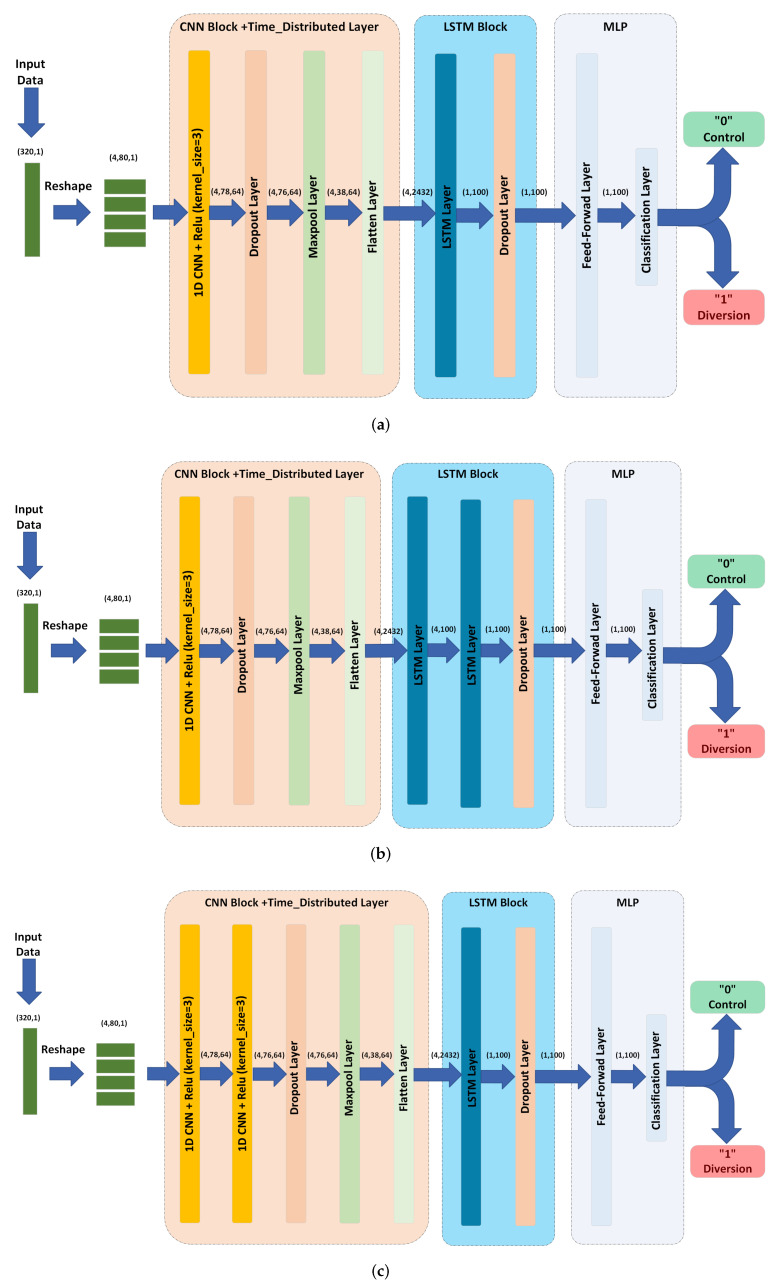
CNN-LSTM based models’ architectures. (**a**) CNN-LSTM model. (**b**) CNN-(2-LSTM) model. (**c**) (2-CNN)-LSTM model.

To exploit both the shape and the time dependencies of the input signal, models with CNN and LSTM layers were examined. Theoretically, CNN can be used to extract shape features, which can be then fed to the LSTM layer to combine the temporal information with shape features to produce global features. For those reasons, we considered three CNN-LSTM based models: CNN-LSTM, CNN-(2-LSTM), and (2-CNN)-LSTM) models. The proposed DNNs take the processed correlation signals obtained from the previous stage as an input and classifies the pipe into control or diversion based on this input. As shown in Figure 10, the proposed neural networks consists of three main blocks, namely, a CNN block, an LSTM block block, and a multi-layer perceptron block. In this model, we are using a time-distributed layer on top of the CNN block in order to preserve the time dependencies of the extracted features from CNN block, which are then fed to the LSTM block as explained next.

In the proposed method, we use a two-step procedure to learn features from the obtained correlation envelope signal. The first is to learn short time-scale features from segments of the correlation envelope signal using a CNN. The second is to learn long time-scale correlations between segments of the correlation envelope signal using a LSTM network.

To learn short time-scale features using a CNN while preserving longer time-scale information related to the temporal variations of the signal, the correlation signal, a 320×1 vector, is split into multiple segments. Each segment is fed into a CNN in parallel (using a time-distributed layer). Then the outputs of the CNNs are downsampled and converted into a time-sequence of feature vectors.

As explained in Section 3, the input of the DNN is a vector with 320 dimensions. This vector is then segmented into $N_{s}$ non-overlapping vectors, each of size $T_{s}\times 1$, where $T_{s}=\frac{320}{N_{s}}$ and $N_{s}$ is a divisor of 320. This process helps in capturing the temporal information in the signal, while decreasing the computational complexity of the model. Note that features will be extracted from each segment separately instead of the whole 320 samples, as we explain next. The final input size after segmentation is $N_{s}\times T_{s}\times 1$. In this work, we select $N_{s}=4$ and $T_{s}=80$. These values are selected after trying different values of $N_{s}$ and selecting the one that leads to the highest accuracy.

Two 1D CNN layers are used as follows. For the first layer, 64 filters (kernels) of size 3×1 are utilized. By applying these filters to the input (4×80×1), an output of size 4×78×1 is produced for each filter. This procedure generates a 4×78×64 array of features as a result of utilizing 64 filters. This output is then fed to a rectified linear unit (ReLU) activation layer.

The output of the first CNN layer is further processed by feeding it to another CNN layer. The second CNN layer operates similar to the first one. We use 64 distinct filters of size 3×64 and a ReLU activation layer to obtain a 4×76×64 output array. The output is then passed to a dropout layer (ratio of 0.5), maxpooling layer (of size 2 and stride 2) and flatten layer. The output of the flatten layer is with a shape of 4×2432, which is then used as an input to the LSTM block.

Note that increasing the number of segments $N_{s}$ leads to increasing the length of this sequence, in return for shorter segments and less features. For instance, for $N_{s}=8$, the length of each segment will be 40, and the result after the flatten layer will be a sequence of 8 vectors with 1152 features each. In general, one should examine different choices for a given application, and select the one that produces the desired accuracy.

In order to extract 100 features from a 4×2432 dimensional input time-series, we employ an LSTM layer composed of 100 LSTM cells. The resulting output has the dimension of 1×100. A dropout layer with a 0.5 dropout ratio is used to regularize the LSTM. The output of the LSTM is then fed into multilayer perceptron networks, which is explained next.

### 5.4. Multilayer Perceptron

The multilayer perceptron is a fully connected dense layer followed by a classification layer. We use multilayer perceptron with 100 inputs, 100 hidden units with ReLU activation functions, and 1 output with a sigmoid activation function. The advantage of a dense layer is that it learns combinations of features from the 100 inputs produced by the LSTM layer. The output of this layer is a probability value between 0 and 1, representing the class of the input sample. This probability is used to determine a class by thresholding at 0.5.

## 6. Experimental Protocol and Results Discussion

In this section, we first provide the experimental protocols, discussing the models’ evaluation metrics. Then we examine and discuss the performance of the proposed algorithms according to the performance metrics presented in Section 6.1.

### 6.1. Experimental Protocols

To evaluate the proposed deep learning models, we compare the performance of the LSTM, 2-LSTM, CNN, 2-CNN, CNN-LSTM, CNN-(2-LSTM), and (2-CNN)-LSTM models (cf. Section 5). We consider different performance metrics to examine the performance of the considered deep learning models. In particular, the considered DNNs are all evaluated using the following metrics:

- Accuracy: The accuracy is the percentage of data points that are classified correctly by the algorithm over the total number of data points. Mathematically, the accuracy can be expressed as
  (9) $$\begin{array}{c} \mathrm{Accuracy}=\frac{T_{P}+T_{N}}{T_{P}+F_{P}+T_{N}+F_{N}}, \end{array}$$
  where $T_{P}$ is the number of the correctly classified positive points (diversions), $F_{P}$ is the number of misclassified negative points (control), $T_{N}$ is the number of correctly classified positive points, and $F_{N}$ is the number of misclassified negative points.
- AUC: The area under the receiver operating characteristic (ROC) curve (AUC) is a metric that measures the ability of a classifier to distinguish between classes across all possible classification thresholds. Therefore, it measures the performance of a model irrespective of the classification threshold.
- Precision: If it is required to evaluate a model only on classifying the positive samples, precision is used. Precision is the ratio of positive samples classified correctly to total positive samples, which can be expressed as
  (10) $$\begin{array}{c} \mathrm{Precision}=\frac{T_{P}}{T_{P}+F_{P}}, \end{array}$$
- Recall: The recall describes the ratio of correctly classified positive samples over the total number of the positive samples, which is given by
  (11) $$\begin{array}{c} \mathrm{Recall}=\frac{T_{P}}{T_{P}+F_{N}}, \end{array}$$
- F1-score: F1-score is the harmonic mean of the precision and the recall values, which can be expressed as
  (12) $$\begin{array}{c} F1-\mathrm{score}=2\times \frac{\mathrm{Recall}\times \mathrm{Precision}}{\mathrm{Recall}+\mathrm{Precision}} \end{array}$$

We calculated these metrics to evaluate the models and specify the model that achieves the highest performance. Furthermore, we studied these models in terms of their convergence rate (see Section 6.2).

Using the aforementioned data-collection procedures, we collect 2292 samples and split them into training, validation, and testing sets, where the splitting ratios are selected as 70%, 10%, and 20%, respectively. We shuffle the samples of the dataset before splitting to guarantee that each subset represents the true distribution of the whole dataset. A full description of the dataset is presented in Table 1. The dataset includes measurements from two sensors. To evaluate the performance of the proposed models, we trained each model with a single sensor reading and then again with both sensors’ readings. Thus, we can evaluate the robustness and performance of each model under a variety of circumstances.

We used Python supported by TensorFlow, a machine learning library, to build and train the considered DNNs. We adopted the categorical cross-entropy as a cost function, and used the ADAM optimizer (a first-order gradient-based approach) to tune the network parameters. To speed up the training process, we used the mini-batch learning approach, where the training dataset was divided into sub-sets (mini-batches) of 64 and fed into the DNN models in an iterative manner. The number of times the training iterates over all the mini-batches is the number of epochs which we set to be 25. Note that these hyperparameters are selected based on trying various values and selecting the ones that lead to the best performance on the validation set. The performance metrics of the DNNs were calculated using the testing set by training the model 25 times (folds) with random initialization and then taking the average and the standard deviation of each metric.

### 6.2. Discussion of the Results

**Figure 11:**
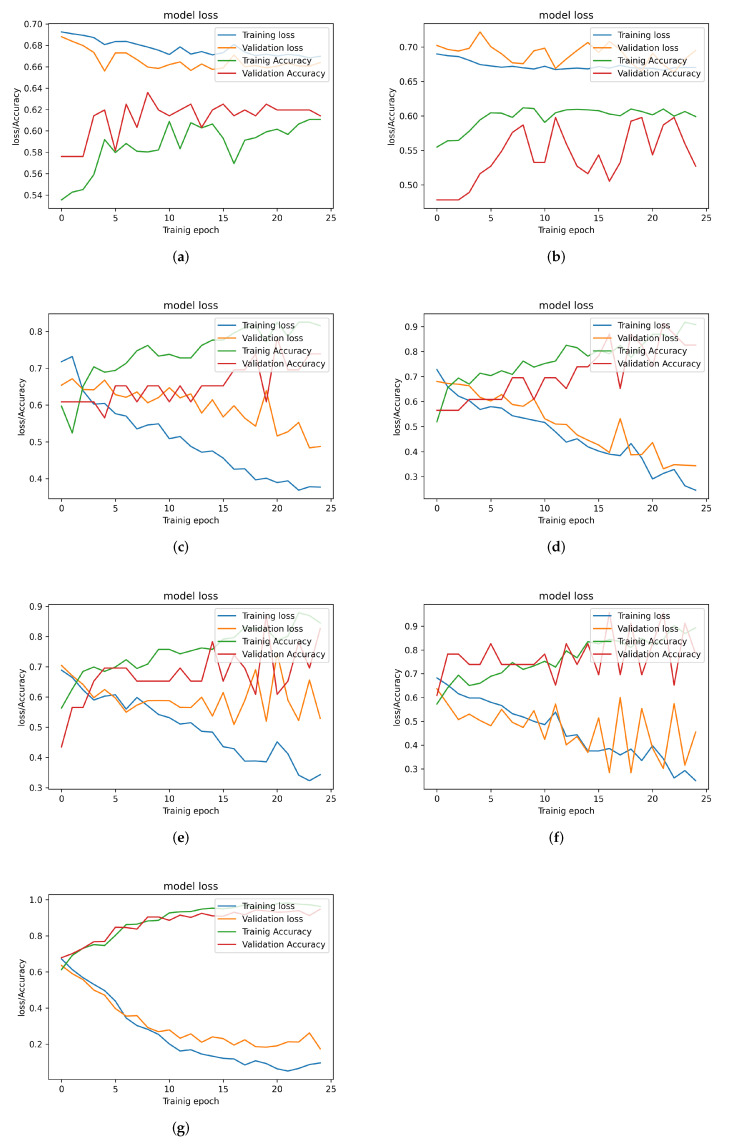
Training and validation loss and accuracy for different trained models (using 1 Sensor reading. (**a**) LSTM model. (**b**) 2 Stacked-LSTM model. (**c**) CNN model. (**d**) 2 Stacked-CNN model. (**e**) CNN-LSTM model. (**f**) CNN-(2LSTM) model. (**g**) (2CNN)-LSTM model.

The results in Table 2 and Figure 11a,b show that the LSTM-based models (LSTM and 2-LSTM models) perform poorly in diversion classification compared to the other models. This is due to the nature of the input signal, which contains long-term time dependencies that the LSTM layer cannot capture due to its limited memory, despite the fact that LSTM networks have superior memory to RNN networks.

In general, Table 2 reveals that the performance of the CNN-based models is better than the performance of the LSTM-based models. Figure 11c,d depict the learning curves of the CNN-based models. The figures shows a steady improvement in both loss and accuracy with increasing the number of epochs. This indicates that the CNN layers are able to extract features from the correlation signal that can distinguish between the diversion and control samples with an average classification accuracy of 83%.

Table 2 also shows that adding the LSTM layer to CNN-based models does not necessarily improve the classification performance when two sensors are considered. However, the table shows that the model (2-CNN)-LSTM model reports the best performance over all the other models. Figure 11g confirms the superiority of the (2-CNN)-LSTM model by showing its fast convergence compared to the other models. These results indicates that an appropriate combination of CNN and LSTM layers may provide a DNN model that can significantly outperform the LSTM- and the CNN-based models.

In Table 2, results show that the highest accuracy reported among all the proposed models is 90.3%. This is because using only one sensor to read the data might not be enough to detect the diversions due to the fact that the sensor orientation may not be suitable to capture the reflected signal from the diversion. While this can be be addressed by considering a more sophisticated DNN architecture, this increases the model complexity, which is not desired in practice. Instead, adding another sensor can help DNN models in characterizing the reflected signals with higher confidence while decreasing complexity as is discussed next.

**Figure 12:**
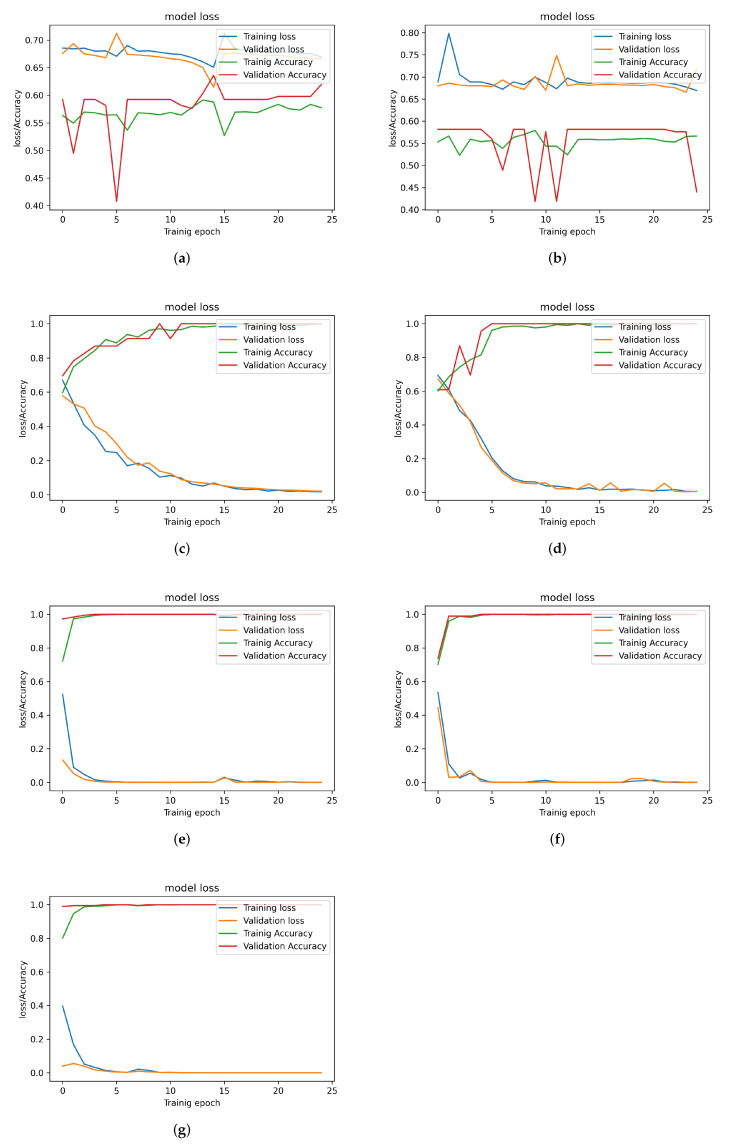
Training and validation loss and accuracy for different trained models (Using 2 Sensors’ readings. (**a**) LSTM model. (**b**) 2 Stacked-LSTM model. (**c**) CNN model. (**d**) 2 Stacked-CNN model. (**e**) CNN-LSTM model. (**f**) CNN-(2-LSTM) model. (**g**) (2-CNN)-LSTM model.

Table 3 shows the performance of all examined models when two sensors are used, and their learning curves are shown in Figure 12. It can be observed that the performance of the LSTM-based models does not improve with adding a sensor to the system due to the difficulty in extracting long-term time dependencies. On the other hand, CNN- and CNN-LSTM-based models all report a significant improvement in all the metrics recorded. This suggests that with enough sensors, the correlation envelope signals become easier to classify, and high accuracy can be achieved, even with a single CNN layer, which is desirable due to its lower complexity. The (2-CNN)-LSTM model shows the best performance, achieving an accuracy of 99.6% when two sensors readings are used.

The proposed scheme can be utilized to be used with different type of pipes. It can also be used to classify different types of defects as long as the defect is captured by the correlation envelop signal.

The limitations of this scheme are as follows. First, the maximum pipe length used in our experiments was 5 m, which raises a question of whether the proposed methods can detect a diversion in longer pipes. It is expected that the accuracy might decrease with increasing the pipe’s length; however, enriching the dataset via using more than two sensors may improve performance. In addition, the performance of the proposed method is expected to deteriorate if the pipes are buried below ground. Therefore, in our future work, we will extend this work to study the diversion detection in buried pipes.

## 7. Conclusions

The method proposed in this paper was capable of classifying HDPE pipes into two control and diversion pipes with an accuracy of 90.3% (when one sensor is used) and 99.6% (when two sensors are used). The method relies on using Zadoff–Chu sequences which are well-known for having excellent autocorrelation properties suited for channel estimation applications. As a result, using signal processing at the receiver combined with a DNN, we were able to determine whether the pipe has a diversion or is a control pipe. It is possible to enhance the algorithm further by adding a second step that estimates the location of the diversion using another DNN, which is left for future work.

## Figures and Tables

**Table 1:** Training, validation and testing sets description.

Dataset	Control	Diversion	Total
Training	718	886	1604
Validation	104	125	229
Testing	184	275	459

**Table 2:** Performance metrics of the tested models (using one sensors’ readings).

Model	Performance Metrics				
	Accuracy	Recall	Precision	F1-Score	AUC
*LSTM*	0.655 (±0.021)	0.845 (±0.036)	0.708 (±0.017)	0.767 (±0.004)	0.637 (±0.001)
*2-LSTM*	0.638 (±0.0242)	0.909 (±0.04)	0.627 (±0.025)	0.74 (±0.003)	0.627 (±0.048)
*CNN*	0.821 (±0.04)	0.921 (±0.04)	0.79 (±0.064)	0.85 (±0.026)	0.915 (±0.02)
*2-CNN*	0.837 (±0.0167)	0.89 (±0.036)	0.83 (±0.038)	0.85 (±0.01)	0.92 (±0.009)
*CNN-LSTM*	0.801 (±0.03)	0.822 (±0.13)	0.82 (±0.061)	0.81 (±0.05)	0.890 (±0.01)
*CNN-(2-LSTM)*	0.822 (±0.04)	0.77 (±0.12)	0.91 (±0.05)	0.82 (±0.06)	0.920 (±0.006)
*(2-CNN)-LSTM*	0.903 (±0.02)	0.9014 (±0.06)	0.905 (±0.04)	0.921 (±0.025)	0.926 (±0.006)

**Table 3:** Performance metrics of the tested models (using two sensors readings).

Model	Performance Metrics				
	Accuracy	Recall	Precision	F1-Score	AUC
*LSTM*	0.544 (±0.037)	0.817 (±0.291)	0.58 (±0.150)	0.61 (±0.104)	0.562 (±0.067)
*2-LSTM*	0.58 (±0.0182)	0.891 (±0.197)	0.64 (±0.121)	0.71 (±0.064)	0.6185 (±0.118)
*CNN*	0.991 (±0.007)	0.9991 (±0.002)	0.995 (±0.004)	0.999 (±0.0002)	0.995 (±0.004)
*2-CNN*	0.997 (±0.003)	0.997 (±0.003)	0.998 (±0.003)	0.998 (±0.002)	0.997 (±0.0094)
*CNN-LSTM*	0.996 (±0.00065)	0.992 (±0.007)	0.998 (±0.001)	0.999 (±0.0004)	0.998 (±0.0045)
*CNN-(2-LSTM)*	0.993 (±0.00130)	0.994 (±0.008)	0.993 (±0.002)	0.992 (±0.00098)	0.994 (±0.0031)
*(2-CNN)-LSTM*	0.996 (±0.001)	0.998 (±0.006)	0.998 (±0.0023)	0.999 (±0.00003)	0.999 (±0.0023)

## Data Availability

Not applicable.
